# Influence of Ni^2+^ on urease activity produced by biofilms of *Arthrobacter oxydans* 1388

**DOI:** 10.1080/13102818.2014.908507

**Published:** 2014-07-08

**Authors:** Dessislava Marinkova, Lyubov Yotova, Jean-Marie Ringeard, Pascal Griesmar

**Affiliations:** ^a^ Department of Biotechnology, University of Chemical Technology and Metallurgy, Sofia, Bulgaria; ^b^ Université de Cergy-Pontoise, ENS, UMR CNRS 8029, Systèmes et applications des technologies de l'information et de l'énergie (SATIE), F-95000, Cergy-Pontoise, France

**Keywords:** biofilms, urease activity, micro-organisms, carrier, *Arthrobacter oxydans*

## Abstract

New TiO_2_-based hybrid materials composed of an organic polymer, cellulose acetate butyrate and copolymer of acrylonitrile acrylamide (AN + AA) were prepared. The effectiveness of immobilization of microbial strain *Arthrobacter oxydans* 1388 on the newly synthesized hybrid membranes was investigated by biochemical methods. The obtained results revealed that the matrix more suitable for biofilm formation was composed of organic polymers without a metal component in the membrane composition. The influence of Ni^2+^ on urease activity produced by biofilms was investigated. The experimental results demonstrated that 2 mg L^−1^ concentration of Ni^2+^ in the nutrient medium is more appropriate for biofilm proliferation.

## Introduction

*Arthrobacter oxydans* has been shown to have a high potential for bioremediation, with high urease activity and urea degradation in wastes.[1,2] *A. oxydans* can detoxify chromium by either reduction or accumulation inside the bacteria and biosorption of chromium (IV) (CrVI) on their surface, and efflux pump.[3,4] *A. oxydans*, when contaminated with high levels of Ni and U, indicates a selection pressure from Ni and shows a capability to utilize hydrocarbons as a nutrition and energy source in a broad range of pH values.[5]

A biotrickling filter with biofilms can achieve high efficiency of NH_3_ and H_2_S removal from waste gases.[6] Granular activated carbon inoculated with *A. oxydans* CH_8_ for NH_3_ removal and *Pseudomonas putida* CH_11_ for H_2_S removal can be used as packing material. Under these conditions, in which 100% H_2_S was removed, extensive tests to eliminate high concentrations of NH_3_ emissions, including removal characteristics, removal efficiency and removal capacity of the system, show promising results.[6]

The aim of this work was to study biofilm formation by *A. oxydans* cells on new hybrid titanium-based matrixes with incorporation of organic polymers, cellulose acetate butyrate (CAB) and a copolymer of acrylonitrile acrylamide (AN + AA), to an inorganic network. The dynamics of the protein, polysaccharide production and urease activity of the biofilm were examined.

## Materials and methods

### Chemicals

Urea, potassium and sodium phosphates and the other salts used were obtained from Merck (Germany). Glucose and bovine serum albumin were obtained from Fluka (Switzerland). All other chemicals were of reagent grade or better.

### Cell culture and biofilm formation

*A. oxydans* strain 1388 from the National Collection for Industrial and Cell Cultures, Bulgaria, was cultured on a solid agar medium containing glucose, yeast extract, peptone and NaCl at 28 °C for 48 h. After incubation, colonies were picked and suspended in a mineral salt medium with a glucose concentration of 10 g L^−1^. The composition of the nutrient medium was: Na_2_HPO_4_.12H_2_O – 9 g L^−1^, KH_2_PO_4_.7H_2_O – 2 g L^−1^, MgSO_4_.2H_2_O – 0.7 g L^−1^, CaCl_2_.2H_2_O – 0.02 g L^−1^, H_2_NCONH_2_ – 0.3 g L^−1^, FeCl_3_.6H_2_O – 0.3 g L^−1^, Na_2_MoO_4_.2H_2_O – 0.1 g L^−1^, H_3_BO_3_ – 0.4 g L^−1^ and CaCl_2_.2H_2_O – 1.34 g L^−1^.[2] After 48-h incubation in a bath shaker at 28 °C, pH 7.2, the cells were suspended in the same nutrient medium containing different concentrations of Ni^2+^ under the same conditions.

The matrices that were used in the experiments for the formation of biofilm are polymeric carriers of three different kinds of polymers and a mixture of polymers with incorporation of the TiO_2_-based part into a hybrid network with a granulated shape.

The copolymer of AN with AA was dissolved in dimethylformamide (DMF) to a concentration of 10% and was further used for granulating. The granulating procedure was performed by dropping a polymer solution into water:methanol (3:1[v/v]) containing 1% NaCl. The stability of granules was achieved by dispersing them in distilled water for 30 min, followed by heating to 85°C for 45 min. The copolymer was prepared in the form of porous granules, 1.5 mm in diameter. The granules were further dried and heated to 70 °C for 3 h. The polymeric membranes were prepared from a copolymer of AN and AA containing 15% acrylamide units and synthesized by radical precipitation copolymerisation.[7]

The polymer matrices used for biofilm formation were a mixture of the AN + AA copolymer and cellulose acetate butyrate (CAB) in a 20:1 ratio.

Precisely, 2.5 mL of a 10% solution of CAB in DMF and 0.125 mL of poly(AN-co-AA) (10% solution in DMF) were mixed by stirring (solution A). Separately, 1 mL of BuOH and 1 mL of Ti(OBu)4 (TBOT) were mixed by stirring (solution B). Subsequently, 0.25 and 0.50 mL of solution B were added dropwise to solution A. During the mixing step, a slight yellow solution is obtained (Ti 1.22% and 2.44% in weight, respectively). After approximately 11 h, gels are obtained.[8]

The obtained matrices were placed in the cell suspension with nutrient medium and biofilms were formed by cell adhesion. The binding of cells was carried out at pH 7 at a temperature of 28 °C under continuous stirring in a bath shaker (220 r min^−1^). The formation of biofilm was tested at 24, 48, 72, 96 and 120 h. After 48 h, the matrices were washed by 0.9% NaCl solution and suspended in a fresh nutrient medium.

### Analytical procedures

The absorbance of the biomass of free cells and that produced by biofilms was measured at 600 nm with a Perkin-Elmer Lambda 2 spectrophotometer (Germany). The renovation of the biofilm was monitored microscopically as well as by means of the turbidity (OD-600) of the effluent. Cell growth of the suspended and immobilized cells was also determined as dry cell weight, according to the method described by Mallette et al. [9] The samples were dried until they reached a constant weight at 105°C. The analysis was checked by the determination of the protein content, using a modification of the Lowry method [10] according to Raunkjær et al.[5] The polysaccharides content was measured using the anthrone method.[11]

### Enzyme assays

Urease activity of free and immobilized cells was determined according to Melnyk.[12] The enzymatic reaction was carried out in a batch vessel with a magnetic stirrer. The reaction was stopped by chilling the mixture in a 2 °C bath after 15 min. The ammonia concentration was measured spectrophotometrically at 480 nm with an identical cuvette, using the Nessler reagent.

### Biosorption of Ni^2+^ by free cells and biofilm

*A. oxydans* 1388 free cells or biofilm were cultured in a liquid nutrient medium containing different concentrations of Ni^2+^ (1.5, 2 and 2.5 mg L^−1^). In order to detect the biosorption of Ni^2+^, samples of 10-mL volume were taken at certain periods of time and were submitted for testing at the Central Scientific Research Laboratory (CSRL), UCTM, Bulgaria, using a high dispersion ICP-OEE spectrometer ‘Prodigy’ (Teledyne Leeman Labs). The residual content of Ni^2+^ was monitored on its basis and the quantity of the accumulated nickel ions was calculated.

## Results and discussion

### Kinetics of extracellular protein production by biofilms

**Figure 1.**
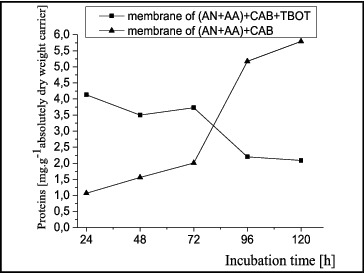
Kinetics of protein production by *Arthrobacter oxydans* 1388 biofilms formed on matrices: (AN + AA) + CAB and (AN + AA) + CAB + TBOT.

After the culture was developed and a biomass was accumulated, polymer carriers were added to the cell suspension to form biofilms. The kinetics of protein and polysaccharide production from the biofilms formed on two different types of polymer carriers was tracked over 120 h, where the 24th hour was assumed as the initial point for the incubation period. The quantity of proteins synthesized from biofilms of *A. oxydans* 1388 on different types of matrices is shown in Figure 1.

The results showed that a larger quantity of proteins were produced from biofilms formed on porous polymer granules of the AN + AA copolymer with CAB. Until 72 h of incubation, a gradual increase in the quantity of the produced proteins was observed, followed by a significant rise to 96 h, and then the production again became gradual, reaching 5.8 mg g^−1^ at 120 h.

The concentration of proteins from the biofilm formed on a matrix containing a metal component in its composition (AN + AA) + CAB + TBOT decreased gradually at 48 h, followed by a significant decrease of the quantity of proteins after 72 h. The figure shows that, in terms of produced proteins, the polymer matrix without a metal component is a better carrier for biofilm formation.

### Kinetics of polysaccharide production by biofilms

**Figure 2.**
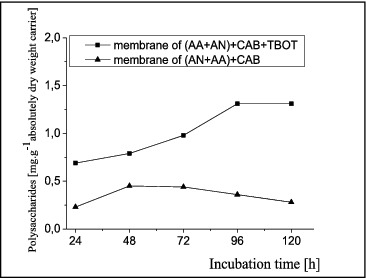
Kinetics of polysaccharides production by *A. oxydans* 1388 biofilms formed on different matrices: (AN + AA) + CAB and (AN + AA) + CAB + TBOT.

The kinetics of polysaccharide production from the formed biofilms is presented in Figure 2.

The figure shows that the quantity of polysaccharides produced from biofilms formed on a matrix of (AN + AA) + CAB + TBOT was larger. For biofilms formed on a matrix containing a metal component, the results showed a gradual increase in the concentration of the synthesized polysaccharides up to 96 h, followed by retention until the end of the incubation period. The maximum peak was observed at 96 g, about 1.3 mg g^−1^.

The biofilms formed on a polymer carrier without titanium butoxide produced much less polysaccharides. A maximum was observed at 48 h (about 0.5 mg g^−1^), but this maximum was approximately three times lower than the maximum peak for the biofilm formed on the TBOT-containing matrix. After 48 h, a continuous decrease in the produced saccharides was observed until 120 h.

### Kinetics of biomass and urease activity in free cells and biofilms

**Figure 3.**
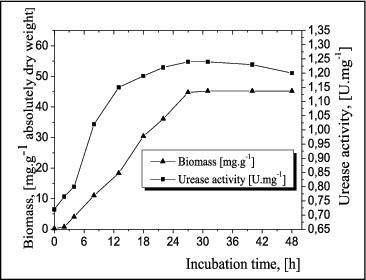
Kinetics of biomass and urease activity in free cells of *A. oxydans* 1388.

The urease activity in free cells of *A. oxydans* 1388 was investigated after the development and accumulation of biomass (Figure 3). Urease activity in free cells was detected as early as 2 h of incubation and was growth dependent. It increased gradually to a maximum of 1.24 U mg^−1^at 28 h, and then slowly began to decrease.

**Figure 4.**
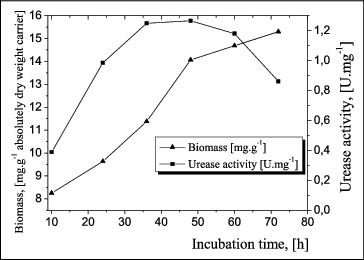
Kinetics of cell growth and urease activity in biofilm formed on an (AN + AA) + CAB copolymer.

To study the kinetics of biomass and the urease activity in biofilms, after growth and accumulation of biomass, polymer carriers of AN + AA copolymer with CAB were added to bind the bacterial cells through adhesion. When the biofilm was formed, it was washed with 0.9% NaCl solution and suspended in a fresh nutrient medium. The urease activity of the cells was detected after the 10 h of cultivation, when the culture was in its exponential phase of development (Figure 4). The highest activity was observed at 36–48 h of cultivation, with a maximum of 1.22 U mg^−1^ at 48 h, followed by a gradual decrease and a sharp decrease of activity after 60 h.

The figures clearly show that the difference between the maximum values of the urease activity under the conditions of a formed biofilm and free cells of *A. oxydans* 1388 is insignificant.

The nutrient medium for cultivating *A. oxydans* 1388 contains urea, which is decomposed by urease to ammonia cations and carbon dioxide. The obtained ammonia cations are product inhibitors of the urease activity and their accumulation influences the rate of the enzyme-catalysed reaction.

The urease activity of the free cells and biofilms of *A. oxydans* 1388 was determined in a nutrient medium containing a standard concentration of Ni^2+^ (2 mg L^−1^). The present study examined the influence of the Ni^2+^ concentration in the nutrient medium with a view to selecting the best concentration of the metal ions for increasing the urease activity in the studied strain.

### Biosorption of Ni^2+^ from free cells and biofilms

**Figure 5.**
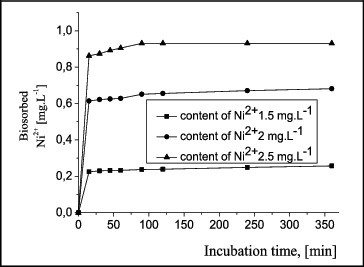
Ni^2+^ biosorption by free cells of *A. oxydans* 1388.

**Figure 6.**
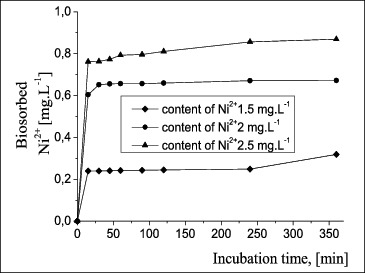
Ni^2+^ biosorption by *A. oxydans* 1388 biofilms in a medium with different Ni^2+^ concentrations.

The results from the biosorption of nickel ions by *A. oxydans* 1388 free cells and biofilms are presented in Figures 5 and 6 for a 6-h cultivation process. The experiments lasted for 48 h and it was shown that the reaction occurred up to the 6th hour.

The changes in the quantity of Ni^2+^ biosorbed by free of *A. oxydans* 1388 cells (Figure 5) showed that the biosorption took place in the first 15 minutes of the incubation period, then the process slowed down, with retention and complete inhibition at a concentration of 2.5 mg L^−1^. The results shown in Figure 6 indicate that the application of biofilms is preferable to free cells, as biofilms were clearly more resistant to the influence of Ni^2**+**^ in a liquid nutrient medium and accumulated metal ions to a greater degree. The reaction took place mainly in the first minutes of the incubation period, and then retention was observed to 4 h at a concentration lower than the initial one, followed by biosorption to 6 h of cultivation. At a higher concentration (2.5 mg L^−1^), the quantity of accumulated ions reached the highest value (34.7%).

The obtained results imply that Ni^2+^ is not sufficiently biosorbed from *A. oxydans* 1388, but the presence of Ni^2+^ ions in the nutrient medium of the studied strain is absolutely necessary for the formation of urease activity.

### Growth kinetics of the biomass of free cells in a medium containing Ni^2+^

**Figure 7.**
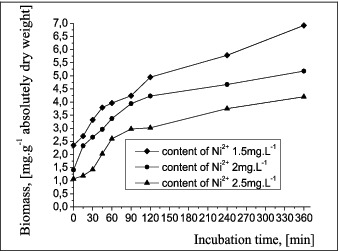
Growth of free cells of *A. oxydans* 1388 in a medium with different of Ni^2+^ concentrations.

The biomass growth of free cells in a medium with different concentrations of Ni^2+^ was monitored over a period of 6 h (Figure 7). Biomass growth was observed until the end of the incubation period, which indicates that the concentration of Ni^2+^ does not significantly influence the accumulation of cell biomass. At a Ni^2+^ concentration higher than the initial one in the nutrient medium, the biomass accumulation process slowed down slightly.

Recent investigations have shown that bacteria such as *A. oxydans* can be considered as metal-tolerant micro-organisms. For example, in uranium deposits located in North America, some new *Arthrobacter* and *Bacillus* sp. strains that have an extremely high ability to accumulate uranium have been discovered.[13] The amounts of uranium accumulated using *Arthrobacter* sp. US-10 depended on the pH of the solution and increased as the external uranium concentration increased.[13]

Other experiments have shown that interactions among radionuclides and microbes are important factors affecting metal mobility in the subsurface. At sites where the concentration of metal contaminants can reach toxic levels, the metal resistance of indigenous microbial populations serves as a critical parameter for the success of *in situ* remediation efforts. Assessment of the uranium resistance of *Arthrobacter* sp. has been carried out by culturing the bacterial cells in various concentrations of uranium (VI) (0.5–57 ppm), showing that due to its unique irregular surface structure, this strain can accumulate more than 90% of uranium.[14]

Metal ion accumulation from microbial cells and the application of biotechnological methods can thus help in the development of alternative methods for detoxication and purification of industrial contaminants. For these innovative methods to be applied, a detailed investigation of the studied strain, as well as of a particular metal biosorption, is necessary.

## Conclusions

In this study, the kinetics of biofilm growth of *A. oxydans* 1388 and the kinetics of production of the two main components of extracellular polymer substances, proteins and polysaccharides, were studied. It was shown that the matrix formed of (AN + AA) + CAB is more suitable for biofilm formation. A slightly higher urease activity was observed in free cells than in biofilms. The experiments for optimization of nickel ion concentration in the nutrient medium showed that the initially chosen concentration of nickel in the nutrient medium (2 mg L^−1^) was the most suitable one. These newly synthesized hybrid membranes are very attractive biomaterials, as they are non-toxic, biocompatible and biodegradable. Further efforts should be made in developing microbial biosensors for sensitive detection of heavy metal pollutants. The use of biosensors has a dynamic trend in recent years and is largely applied because of the sensitivity, selectivity and simplicity of biosensors. They also become more and more a synergetic combination between biotechnology and microelectronics.
